# Temporal interference stimulation of the nucleus accumbens in obsessive‐compulsive disorder

**DOI:** 10.1002/gps3.70059

**Published:** 2026-09-15

**Authors:** Binxin Huang, Yulong Cheng, Huaiyue Cao, Di Li, Dongdong Shi, Zhen Wang

**Affiliations:** ^1^ Shanghai Mental Health Center Shanghai Jiao Tong University School of Medicine Shanghai China; ^2^ Shanghai Intelligent Psychological Evaluation and Intervention Engineering Technology Research Center Shanghai China

**Keywords:** neuromodulation, nucleus accumbens, obsessive‐compulsive disorder, temporal interference stimulation

**Abbreviations**
DBS: deep brain stimulation
MRI: magnetic resonance imaging
DSM‐5: Diagnostic and Statistical Manual of Mental Disorders, Fifth Edition
EEG: electroencephalography

Obsessive‐compulsive disorder (OCD) is associated with dysfunction in cortico‐striato‐thalamo‐cortical and limbic circuits,^1^ which support salience processing, reward learning and compulsive action selection. Among circuit‐informed neuromodulation targets, the nucleus accumbens (NAc) is of particular interest because converging evidence has implicated it in altered reward‐related processing and abnormal frontostriatal connectivity in OCD.^2^, ^3^, ^4^ In treatment‐refractory OCD, bilateral NAc deep brain stimulation (DBS) has shown clinical benefit,^5^ and mechanistic and connectomic studies suggest that symptom improvement may relate to normalisation of frontostriatal network activity and engagement of shared prefrontal‐subcortical pathways.^6^, ^7^ However, the invasive nature of traditional DBS highlights the need for novel, noninvasive alternatives capable of engaging deep brain structures.

Temporal interference (TI) stimulation remedies this defect. It provides a noninvasive approach for shaping a low‐frequency amplitude‐modulated electric field at depth using interfering kilohertz‐range currents.^8^ Emerging human evidence further supports the capacity of TI‐based approaches to engage striatal circuitry, including under theta‐burst stimulation paradigms.^9^ Because optimal field distributions vary across individuals, individualised optimisation is likely to be important for deep‐target TI applications.^10^

Against this background, we report a patient with OCD who remained symptomatic despite adequate pharmacological and psychotherapeutic treatment and who subsequently received individualised theta‐burst patterned TI targeting the bilateral NAc. This case provides an early clinical example of individualised NAc‐targeted TI in OCD and adds preliminary evidence supporting the feasibility and short‐term symptomatic benefit of this noninvasive, circuit‐informed approach.

This report was approved by the Institutional Review Board of Shanghai Mental Health Center (Approval No. 2025‐47), and written informed consent was obtained. Individualised stimulation montages were derived from structural magnetic resonance imaging (MRI) using the NervioX optimisation algorithm to determine electrode positions and current amplitudes (figure 1A). Stimulation was delivered twice daily for 7 consecutive days, totalling 14 sessions. Each session lasted 40 minutes. We sequentially targeted the left and right NAc for 20 minutes per side. Two sinusoidal carrier currents were applied through separate bipolar electrode pairs. One channel continuously delivered 2000 Hz. During burst windows, the second channel switched from 2000 to 2130 Hz. This frequency offset produced a 130‐Hz amplitude‐modulated envelope. A theta‐burst patterned schedule was used, with 2 s trains repeated every 10 s; within each train, burst events recurred every 200 ms and comprised three cycles of 130 Hz modulation. During interburst and intertrain intervals, the second channel reverted to 2000 Hz, yielding nonmodulated high‐frequency current. Stimulation intensities were individualised based on tolerability. We set the intensity as a fixed proportion of the patient's maximum tolerated threshold. This approach resulted in currents ranging from 1 to 5 mA across the bipolar pairs. Left‐ and right‐sided targeting was achieved by swapping channel assignments. Before each session, the patient underwent standardised symptom provocation for 3–5 minutes. The goal was to reach a Subjective Units of Distress Scale score of at least 40/100. During stimulation, symptom engagement was maintained using intermittent cue exposure. No changes to the intervention protocol were made during the treatment course.

**FIGURE 1:**
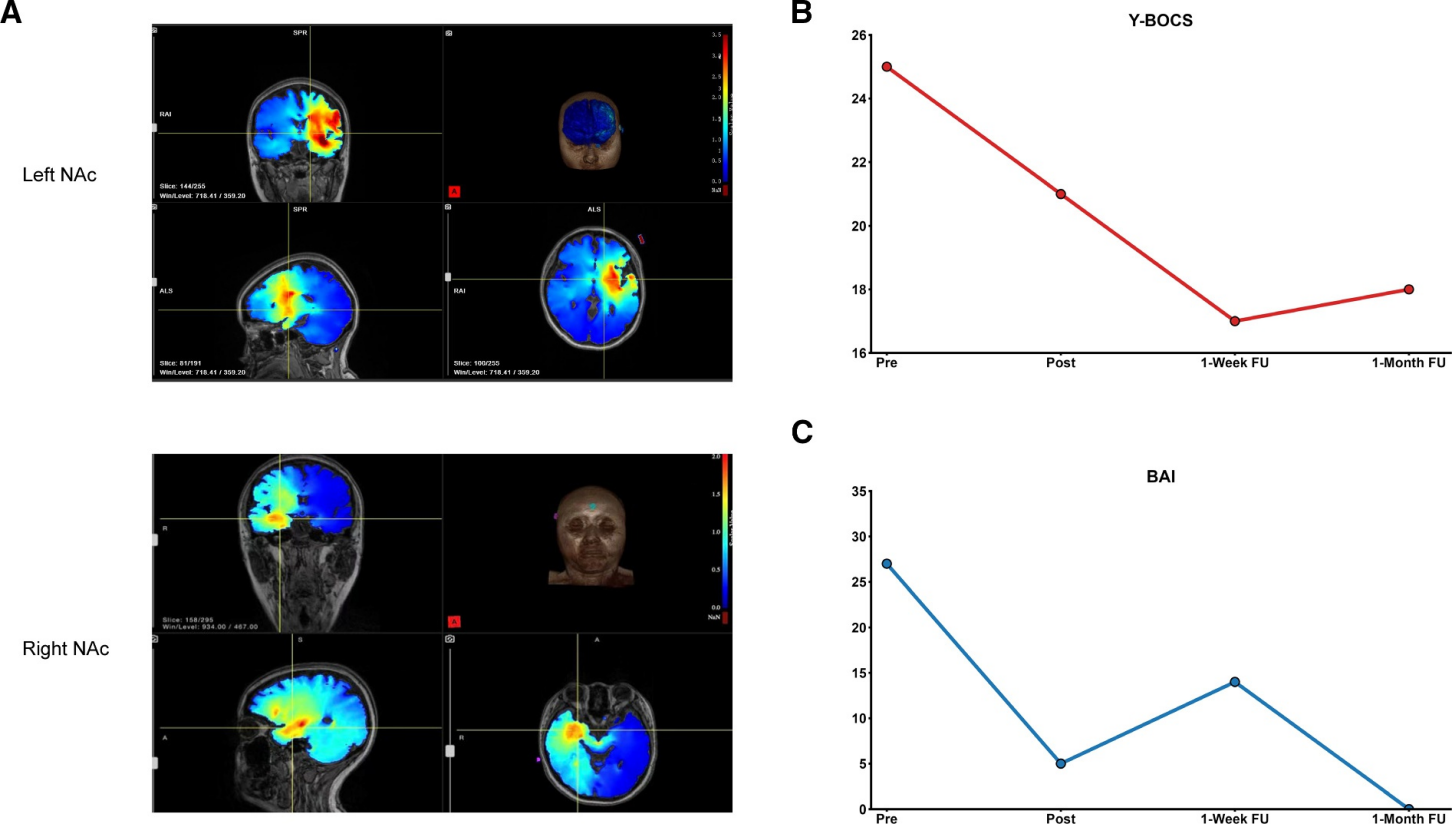
Individualised NAc‐targeted theta‐burst patterned temporal interference stimulation and clinical trajectories in a patient with OCD. (A) Modelled peak 130‐Hz amplitude‐modulated electric‐field envelopes for the individualised left and right NAc montages; the cross hairs indicate the intended targets. (B) Y‐BOCS scores at baseline (pre), end of treatment (post), 1‐week FU and 1‐month FU. (C) BAI scores at the same assessment points. BAI, Beck Anxiety Inventory; FU, follow‐up; NAc, nucleus accumbens; OCD, obsessive‐compulsive disorder; Y‐BOCS, Yale–Brown Obsessive‐Compulsive Scale.

Clinical symptoms were assessed using the Yale–Brown Obsessive‐Compulsive Scale (Y‐BOCS) and Beck Anxiety Inventory (BAI) at baseline (pre), end of treatment (post), 1‐week follow‐up and 1‐month follow‐up. As shown in figure 1B, C, the patient's Y‐BOCS scores changed from 25 at baseline to 21 at post, 17 at 1 week and 18 at 1 month. The corresponding BAI scores were 27, 5, 14 and 0. These changes indicate clinical improvement, with OCD severity decreasing from severe to moderate and anxiety symptoms from severe to mild or minimal. The patient reported subjective improvement in obsessive‐compulsive symptoms and anxiety during follow‐up and completed all 14 planned sessions without interruption or discontinuation due to intolerance. Based on patient self‐report and routine clinical monitoring, no adverse events or unanticipated problems were reported or observed during treatment or follow‐up.

The patient was a 25‐year‐old woman with a 13‐year history of OCD and no documented medical or psychiatric comorbidity. There was no known family history of OCD or other major psychiatric disorders. No relevant lifestyle factors or genetic findings were identified. Physical and neurological examinations were unremarkable. Diagnosis was established according to diagnostic and statistical manual of mental disorders, fifth edition criteria. Her predominant symptoms included contamination concerns, excessive worrying, repetitive cleaning, repeated checking and arranging objects in a fixed order. She had shown inadequate response to at least two adequate trials of serotonin reuptake inhibitors and 20 sessions of psychotherapy. At study entry, she had been receiving sertraline 200 mg/day for at least 8 weeks, and the dosage remained stable throughout treatment and follow‐up.

This case suggests that individualised bilateral NAc‐targeted theta‐burst patterned TI is feasible in OCD and may be followed by short‐term improvement in obsessive‐compulsive symptoms. This report has two novel aspects. First, it used individualised TI to noninvasively target the bilateral NAc, a deep brain structure implicated in OCD, with a theta‐burst pattern. Second, TI was delivered during symptom provocation, with anxiety maintained throughout stimulation. This approach may promote state‐dependent engagement of OCD‐related circuits. However, symptom provocation may also have contributed independently or synergistically to the clinical response. Thus, the improvement cannot be attributed to TI alone. Future controlled studies should compare active and sham TI under identical symptom‐provocation conditions. A factorial design may further clarify the independent and interactive effects of TI and symptom provocation. Several additional limitations should be noted. First, objective neurophysiological or neuroimaging assessments were not performed before or after treatment. Electroencephalography and functional MRI could have provided evidence of target engagement and circuit modulation. Therefore, the neural effects of the intervention could not be determined. Second, clinical follow‐up was limited to 1 month. Consequently, the durability of the observed improvement remains unknown. Given the chronic course of OCD and the treatment‐resistant status of this patient, longer follow‐up is needed to determine whether the clinical effects persist. This case provides preliminary clinical and translational support for further investigation of individualised NAc‐targeted TI in OCD. Larger prospective studies are required. These studies should include appropriate control conditions, systematic safety assessments, longer follow‐up and multimodal outcome measures.

## AUTHOR CONTRIBUTIONS

**Binxin Huang:** writing—original draft, formal analysis, visualization, investigation. **Yulong Cheng:** software, visualization, writing—review and editing. **Huaiyue Cao:** investigation, writing—review and editing. **Di Li:** investigation. **Dongdong Shi:** conceptualization. **Zhen Wang:** supervision, conceptualization, investigation, writing—review and editing.

## FUNDING

This work was supported by funding from the National Natural Science Foundation of China (82230045), the Shanghai Science and Technology Committee (25Y22800100) and the Yangtze River Delta Science and Technology Innovation Community Joint Research (Basic Research) Project (2025CJSZN01700).

## ETHICS STATEMENT

This study was approved by the Institutional Review Board of Shanghai Mental Health Center (Approval No. 2025‐47).

## CONSENT

Written informed consent was obtained from the patient for the publication of this case report and any accompanying images.

## Data Availability

De‐identified data supporting the findings of this report are available from the corresponding author upon reasonable request, subject to institutional and ethical approvals.
